# Impact of RNA Virus Evolution on Quasispecies Formation and Virulence

**DOI:** 10.3390/ijms20184657

**Published:** 2019-09-19

**Authors:** Madiiha Bibi Mandary, Malihe Masomian, Chit Laa Poh

**Affiliations:** Center for Virus and Vaccine Research, School of Science and Technology, Sunway University, Kuala Lumpur, Selangor 47500, Malaysia

**Keywords:** RNA viruses, quasispecies, spontaneous mutations, virulence, plaque phenotype

## Abstract

RNA viruses are known to replicate by low fidelity polymerases and have high mutation rates whereby the resulting virus population tends to exist as a distribution of mutants. In this review, we aim to explore how genetic events such as spontaneous mutations could alter the genomic organization of RNA viruses in such a way that they impact virus replications and plaque morphology. The phenomenon of quasispecies within a viral population is also discussed to reflect virulence and its implications for RNA viruses. An understanding of how such events occur will provide further evidence about whether there are molecular determinants for plaque morphology of RNA viruses or whether different plaque phenotypes arise due to the presence of quasispecies within a population. Ultimately this review gives an insight into whether the intrinsically high error rates due to the low fidelity of RNA polymerases is responsible for the variation in plaque morphology and diversity in virulence. This can be a useful tool in characterizing mechanisms that facilitate virus adaptation and evolution.

## 1. Introduction

With their diverse differences in size, structure, genome organization and replication strategies, RNA viruses are recognized as being highly mutatable [1]. Their high mutation rates make it very difficult for therapeutic interventions to work effectively and very often they develop resistance to antiviral drugs and antibodies elicited by vaccines [2,3]. This poses a real threat to how emerging infectious agents could be prevented or treated [4]. The success of the evolution of RNA viruses arises from their capacity to utilize varying replication approaches and to adapt to a wide range of biological niches faced during viral spread in the host. One of the factors affecting the emergence or re-emergence of infectious diseases is the genetics of the infectious agents [1].

Only about 15 viral diseases can be effectively prevented through FDA approved vaccinations [5]. This attests to the urgency to understand the mechanisms by which viruses can overcome the different pressures applied to restrict their replications. In the last four decades, breakthroughs in molecular biology have favored in-depth analysis of virus isolates. Findings from other studies have suggested that populations of RNA viruses are divergent and favor an active evolution of RNA genomes. The quick evolution of RNA genomes could lead to variant sequences that differ by one or two nucleotides from the wild-type sequence in the population. It is further suggested that each viral RNA population of 10^9^ or more infectious particles was always a mixture of various variants despite being isolated from a single clone [6].

Such heterogeneity within the virus population could be explained by the existence of not a single genotype within the species but rather an ensemble of related sequences known as the quasispecies [7]. Developed by Manfred Eigen and Peter Schuster, the concept of quasispecies was defined as a mutant distribution dominated by a primary sequence with the highest rates of replication between the components of the mutant distribution. The phenomenon of quasispecies was further supported by the “hypercycle” theory as a self-organization principle to include different quasispecies in a higher-order organization that eases evolution into more complicated forms such that the coding capacity and catalytic activities of proteins are taken into account [8,9,10].

Mutants with varying levels of infectivity generated from a mutated gene occurred regularly in a virus population due to the high mutation rates [6]. The error-prone replication ability of RNA viruses and the shorter generation times can be used to explain the variations in evolution rates between DNA and RNA viruses. While mutation rates for DNA genomes have been estimated to be between 10^−7^ and 10^−11^ per base pair per replication [11], the RNA dependent RNA polymerase (RdRp) showed typically low fidelity whereby the mutation rate is of roughly 10^−4^ mutations per nucleotide copied, which is greater than that of almost all DNA viruses [7,12,13]. This characteristic of the RNA polymerase in RNA viruses led to the generation of diverse offspring with different genotypes in shorter generation times.

There is some general consensus regarding quasispecies that have been established. For instance, the presence of diverse mutants in a population of viruses is a reality which can affect the biological behavior of the virus in vivo due to the complexity and amplitude of the mutant spectra [14]. Moreover, interactions amongst variants of a quasispecies population was classified into three types namely—cooperation, interference and complementation. Cooperative interactions arise from those variants exhibiting advantageous phenotypes compared to the wild-type while interfering interactions are those exemplified in variants with detrimental effects on the replication of the virus. However, complementation interactions have no positive or negative effects on the virus population [15].

Considerably less is known of the relationships between the evolution of RNA viruses with respect to virulence. The dynamics of quasispecies has explained the failure of monotherapy and synthetic antiviral vaccine but opened up new avenues for exploration [14]. Specifically, unanswered questions pertaining to quasispecies remain—What are the underlying mutations responsible for long term tenacity compared to those of extinction? Are there any molecular determinants which are the root cause of higher virulence in a quasispecies population?

Frequent occasional outbreaks of emerging and re-emerging viral diseases such as Dengue fever, West Nile Fever, Zika virus disease, Chikungunya disease, Middle east respiratory syndrome, Ebola virus disease and many others have been targets for therapeutic interventions. Long lasting protection against viral infections is best achieved via vaccinations through live attenuated viruses (LAVs). In order to generate stable vaccine strains, the evolution of these viruses must be properly understood. This review is centered on the examination of the evidence for the heterogeneous nature of RNA genomes (quasispecies), the factors leading to quasispecies formation and its implications on virulence.

The phenomenon of quasispecies has been well reported for many viruses belonging to different families and genera [16,17,18]. There is no clear idea whether emerging viruses such as Zika virus, Ebola virus, West Nile virus, Dengue virus and many others owe part of their evolution to higher virulence conferred by the presence of specific quasispecies within the viral population. Exploring the underlying mechanism of virulence stemming from a quasispecies population remains of interest. In this review, we examine reported cases of quasispecies and their implications on virulence.

## 2. Picornaviridae

**Figure 1:**
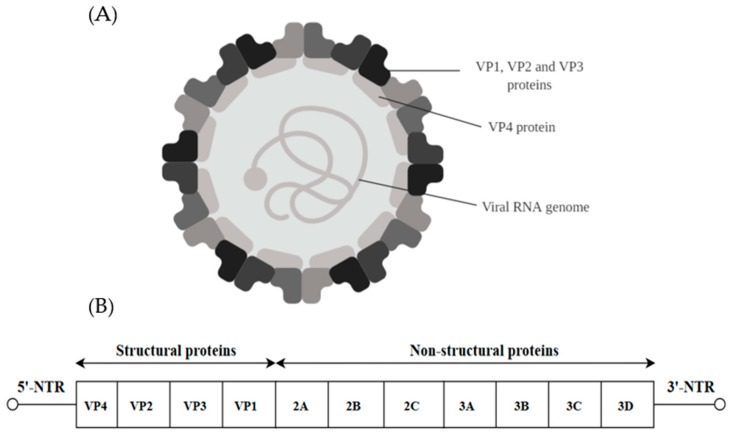
(**A**) The structure of the picornavirus (30 nm). The viral structural proteins (VP1–VP4) are depicted. (**B**) Schematic representation of the EV-A71 genome (7.4 Kb). The Open Reading Frame (ORF) contains the structural viral protein P1 which is cleaved to yield VP1, VP2, VP3 and VP4 and non-structural viral proteins P2 (cleaved to yield 2A, 2B and 2C) and P3 (cleaved to yield 3A, 3B, 3C and 3D). The 3′-NTR end of the genome contains the poly (**A**) tail.

Viruses of the family *Picornaviridae* can be classified into genera such as *Enterovirus*, *Parechovirus*, *Aphotovirus* and others. The virion is made up of a non-enveloped capsid of 30 nm surrounding a core positive stranded ssRNA genome (Figure 1A) [19]. The genome is approximately 7 kb in size and possesses a single long ORF flanked on both ends by the 5′-non-translated region (5′-NTR) and the 3′-non-translated region (3′-NTR) [20]. The 5′-NTR has an internal ribosome entry site (IRES) which controls cap-independent translation. The ORF comprising 6579 nucleotides can be classified into three polyprotein regions, namely, P1, P2 and P3. They encode for structural proteins (VP1 to VP4) in the P1 region and non-structural proteins in the P2 (2A–2C) and P3 regions (3A–3D) following proteolytic cleavage (Figure 1B). The viral capsid proteins VP1, VP2 and VP3 are displayed on the external structures of the EV-A71 viral particle whereas VP4 is found within the internal structures of the capsid [21].

### 2.1. Poliovirus (PV)

Poliovirus is found within the *Human Enterovirus C* species of the *Picornaviridae* family and can be classified into three distinct serotypes (1, 2 and 3). Most poliovirus infections cause an asymptomatic incubation period followed by a minor illness characterized by fever, headache and sore throat which mainly affects children. However, PV infections can lead to paralytic poliomyelitis which can result in death. Following the WHO 1988 polio eradication program, the number of poliomyelitis has been reduced by 99% worldwide but a small number of countries still have sporadic outbreaks of polio [22].

The poliovirus population diversity was evaluated in the brain of the murine model during viral spread. It was observed that only a fraction of the original injected viral pool was able to move from the initial site of inoculation to the brain via the ‘bottleneck effect.’ To determine the maintenance of the quasispecies during infection in vivo, 6–10 weeks old mice were inoculated in the leg with individual viruses. Total RNA recovered from the brain tissues revealed that four viruses were shown to be capable of spreading to the brain with their introduced mutations unchanged. Therefore, it was postulated that the innate immune response reduced the viral pathogenicity by limiting the diversity of viruses during spread to vulnerable tissues [16]. Two mechanisms to explain the bottleneck effect have been speculated, namely—the “tough-transit” model and the “burned-bridge” model. The “tough-transit” model suggests that virus trafficking within the murine model has a low probability of success passing the blood-brain barrier. However, once in the CNS, it acts as a founder virus and re-establishes a population with initial limited diversity. On the other hand, the “burned bridge” model stipulates that it is not tough for the virus to physically reach the blood-brain barrier. Thus, when the first few viruses reach the gateway to the brain, the host innate immune response triggers an antiviral state [16].

In order to verify whether limiting the genomic diversity of a viral population has any effect on its evolution, a study was conducted on a strain of poliovirus with a substitution of Glycine 64 to Serine (G64S) in the RNA polymerase of the virus. The outcome of one-step growth curves and northern blot analysis of genomic RNA synthesis confirmed that the G64S mutation showed greater fidelity without a considerable reduction in the overall efficiency of RNA replication. The study hypothesized that having greater heterogeneity within a viral population allows it to adapt better to changing environments encountered during an infection and indeed the finding showed that boosting the fidelity of poliovirus replication had a noticeable effect on viral adaptation and pathogenicity. The poliovirus strain with a mutated RNA polymerase carrying an altered amino acid residue (G64S) was observed to replicate similarly to the wild-type counterpart but produced lower genomic diversity and subsequently was incapable of adapting well under detrimental growth situations. This study showed that the diversity of the quasispecies was associated with increased virulence rather than selection of single adaptive mutations. Alongside previous observations, these findings indicated a rise in the error rate over the tolerable error threshold which induced viral extinction, suggesting that the rate of viral mutation was precisely modulated and most likely had been finely tuned during the evolution of the virus [7]. It was further revealed that curbing the diversity of a RNA viral population by raising the fidelity of the RNA polymerase had a direct effect on its pathogenicity and capacity of the viruses to escape antiviral immunity [23]. These findings support the fact that RNA viruses have developed minimal viral polymerase fidelity to facilitate quick evolution and adaptation to novel situations [24].

### 2.2. Enterovirus 71 (EV-A71)

EV-A71 belongs to the genus *Enterovirus* within the family of *Picornaviridae*. It was first characterized in 1969 in California, USA [25] and is one of the main etiological agents of hand, foot and mouth disease (HFMD) [26]. Some cases of EV-A71 infections have been associated with neurological complications such as aseptic meningitis, brainstem encephalitis and acute flaccid paralysis [27]. In China, enteroviruses such as EV-A71 and CV-A16 have caused 7,200,092 cases of HFMD between 2008 to 2012. The mortality rate was highest among children below the age of five. It was further reported that 82,486 patients developed neurological complications and 1617 deaths were confirmed by the laboratory to be caused by EV-A71 [28,29].

The course of evolution through which EV-A71 evolves to escape the central nervous system (CNS) was investigated by complete sequencing and haplotype analysis of the strains isolated from the digestive system and the CNS. A novel bottleneck selection was revealed in various environments such as the respiratory system and the central nervous system throughout the dissemination of EV-A71 in the host. Consequently, a dominant haplotype resulting from the bottleneck effect caused a change from viruses harboring VP1–3D to VP1–31G where the amino acid 31 was a favorable site of selection among the circulating EV-A71 sub-genotype C2. VP1–31G was present at elevated levels amongst the population of mutants of EV-A71 in the throat swabs of subjects with severe EV-A71 infections. Furthermore, in vitro studies showed that VP1–31D virus isolates had higher infectivity, fitness and virion stability, which sustained the virus infections in the digestive system. Speculations were that such factors benefitted the virus in gaining added viral adaptation and subsequently enabled viral spread to more tissues. These beneficial abilities could also justify the reduced number of VP1–31D viruses located in the brain following positive selection. The VP1–31G viruses presenting the major haplotype in the central nervous system displayed increased viral fitness and growth rates in neuronal cells. This implied that the VP1–31G mutations aided the spread of the mutant virus in the brain which resulted in serious neurological complications in patients. It was speculated that the fluctuating degree of tissue tropism of EV-A71 at diverse inoculation sites resulted in the bottleneck effect of the viral population having a mutant spectrum. Hence, the adaptive VP1–31G haplotype became dominant in neuronal tissues and once the infection was achieved, VP1–31G viruses expedited bottleneck selection and propagation into the skin and CNS. Among the three minor haplotypes (C to E) which co-existed in various tissues, the minor haplotype C was isolated in the intestinal mucosa and throat swab specimens. The minor haplotype D was isolated from specimens obtained from the respiratory and digestive systems. However, the minor haplotype E appeared in throat swabs and the basal ganglia but not the intestinal mucosa, hence, suggesting that the intestinal mucosa is the initial replication site of the EV-A71. Collectively, these data showed that the EV-A71 quasispecies utilized the dynamic proportion of varying haplotype populations to co-exist, sustained the ability of the population to adapt and enabled the propagation in different tissues. Lastly, the study concluded that the selection of haplotype(s) might be a driving factor in viral dissemination and severity of infections in humans as well as the virulence in EV-A71 infected patients [30].

## 3. Flaviviridae

**Figure 2:**
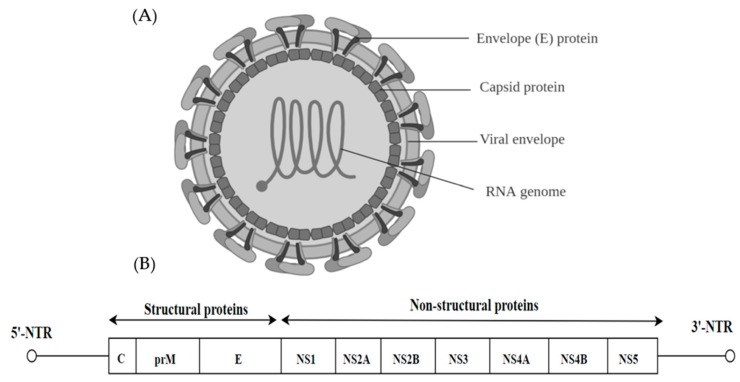
(**A**) The virion of the flavivirus (40–60 nm) is illustrated highlighting the position of the E dimers (E1 and E2), capsid protein and RNA genome (**B**) Schematic representation of the Flavivirus structure and genome (11 kb). The position of the 5′-NTR and the 3′-NTR are shown. The Open Reading Frame (ORF) contains the structural viral proteins (C, prM and E) and the non-structural viral proteins (NS1, NS2A, NS2B, NS3, NS4A, NS4B and NS5).

Of the *Flaviviridae* family (genera *Flavivirus*, *Pestivirus*, *Pegivirus* and *Hepacivirus*), there are 89 animal viruses with a small, positive-sense, single stranded RNA genome [31]. The virions are 40–60 nm in diameter, spherical in shape and contain a lipid envelope (Figure 2A). The majority of these viruses are arthropod-borne and transmitted via infected mosquitoes and ticks. They are considered as emerging and re-emerging pathogens such as dengue virus (DENV), West Nile Virus (WNV), Zika Virus (ZIKV) and these viruses pose a global threat to public health by causing significant mortality [32]. The flaviviral genome is approximately 11 kb and has a single open reading frame (ORF), which is flanked by untranslated regions (5′ and 3′ NTR). The ORF encodes three structural proteins (C, M and E) and 7 non-structural proteins (NS). The non-structural proteins include large, highly conserved proteins NS1, NS3 and NS5 and four small hydrophobic proteins NS2A, NS2B and NS4A and NS4B (Figure 2B) [33].

### 3.1. Dengue Virus (DENV)

Dengue is a re-emerging arboviral infection transmitted by *Aedes* mosquitoes that infect up to 390 million people worldwide annually, of which 100 million infections are symptomatic [34]. The global incidence of dengue has grown dramatically in recent decades and about half of the world’s population is now—at risk [35]. In particular, it represents a growing public health problem in many tropical and sub-tropical countries, mostly in urban and semi-urban areas. The viral etiology of dengue is characterized by biphasic fever, headache, pain in various parts of the body, prostration, rash, lymphadenopathy and leukopenia that affects young children and adults [36]. However, dengue infection can progress to severe dengue hemorrhagic fever (DHF) and dengue shock syndrome (DSS). Severe dengue affects most Asian and Latin American countries and has become a leading cause of hospitalizations and deaths among children and adults in these regions. It is a life-threatening disease with 500,000 cases being admitted to hospitals and 25,000 deaths yearly [37].

A previous study compared a DENV vaccine candidate strain, DENV-3 PGMK30FRhL3 (PGMK30), which produced acute febrile illnesses with another clinically safe DENV vaccine candidate DENV-2PDK53 (PDK53). Using in vitro and in vivo approaches, the infectivity of the two vaccine strains was investigated to assess the molecular determinants of plaque size. It was revealed that the small plaque displayed by the PGMK30 strain in BHK-21 cells was due to its reduced in vitro growth rate. On the other hand, the PDK53 strain which produced the small plaques was observed to grow rapidly but was unable to evade antiviral responses which restricted its ability to spread. The slow growth rates of the two strains were suggested to be due to two different key mechanisms—the growth of PDK53 appeared to be modulated by antiviral responses while PGMK30 was slow to spread to surrounding cells but was able to evade immune detection. It was hypothesized that if the plaque size of PDK53 was hindered by the antiviral response, interfering with its activation by silencing pSTAT1 would be expected to alter the plaque characteristics of the PDK53 but not PGMK30 or the wild-type. In line with the hypothesis, a marked increase in pSTAT1 was shown in cells surrounding the foci of PDK53 infection but no increase was detected in PGMK30 and the wild-type. At least two different mechanisms dictate the plaque phenotype and elucidating the exact mechanism of how it caused the formation of small plaque size is an efficient way to choose future live-attenuated vaccine strains for clinical development [38]. 

From another perspective, differences in the envelope (*E*) gene sequence was investigated using the plasma samples of six DENV infected patients. The first account of viral quasispecies of DENV in vivo was reported using clonal sequencing analysis whereby the simultaneous occurrence of diverse variant genomes was observed. The degree of genetic diversity was revealed to fluctuate among patients with the mean proportion being 1.67%. Moreover, out of 10 clones derived from dengue infected plasma, 33 nucleotide substitutions were detected, of which 30 were non-synonymous mutations. Of particular interest, mutations at amino acid residues 290 and 301 resulted in the presence of two stop codons which indicated that genome-defective dengue viruses (5.8%) were also present within the quasispecies population. It was hypothesized that this might have significant impact on the pathogenesis of the dengue virus [39]. Recently, Parameswaran et al. profiled the intra-host viral diversity of samples from 77 patients via whole-genome amplifications of the entire coding region of the DENV-3 genome. A significant difference in the viral makeup between naïve subjects and patients with DENV-3 immunity revealed that the immune repertoire of the host is responsible for the degree of diversity exhibited by the viral population. Subsequently, identification of the hotspots responsible for the intra-host diversity revealed that few spots were crucial for intra-host diversity. The major hotspots for diversity were revealed in more than 59% of the samples at three codon coordinates—amino acid residues 100 and 101 in the M protein and residue 315 in the AB loop of the E Domain III. The residue E_315_ was speculated to have arisen as an immune escape variant in response to the pressure exerted by the immune defense mechanism. These findings highlighted the importance of host-specific selection pressures in the evolution of DENV-3 viral population within the host and this could eventually lead to the intelligent design of a vaccine candidate identified from the prevalent escape variants such as those bearing the E_315_ [40]. It was reported that within the quasispecies population, amino acid substitutions occurred on the surface of the E protein which was involved in interactions with other oligomers, antibodies and host cell receptors. In particular, two amino acid substitutions at positions E452 and E455 were mapped to the E protein transmembrane domain, E450 to E472, which functioned as the membrane anchor for E protein. Intra-host quasispecies analysis using the *E* gene sequences also identified several amino acids on the surface of the E protein which altered the properties of the virus. The conformational rearrangements that led to the fusion of the virus and the host cell membrane was altered. The amino acids detected in the quasispecies consensus sequence were observed to be less frequent in the E proteins from patients suffering from mild disease than from patients with severe onset of dengue infection. Thus, the quasispecies might harbor specific variants that are crucial for the pathogenesis of the disease [41]. Understanding the significant molecular determinant of pathogenesis through the analysis of quasispecies could lead to the rational design of a DENV vaccine.

### 3.2. Zika Virus (ZIKV)

Zika Virus was first discovered in 1947 when it was isolated from *Aedes Africanus* mosquitoes [42]. It belongs to the *Flavivirus* genus within the *Flaviviridae* family. Zika infections have been reported in Egypt [43], East Africa [44], India [45], Thailand, Vietnam [46], Philippines and Malaysia [47]. 

An Asian/American lineage ZIKA virus (ZIKV) formed 2 types of plaques—large and small. The large plaque variant was observed to have faster growth kinetics compared to the small plaque variant. Sequencing of the plaque variants showed that the large plaque variant had a guanine nucleotide at position 796 (230^Gln^) while the small plaque clone had an adenine at the same position. A recombinant clone carrying the G796A mutation was produced using an infectious molecular clone of the ZIKV MR766 strain. The plaque size produced by the recombinant clone was smaller when compared to the parental strain and its growth rate was significantly reduced in Vero cells. In vivo studies demonstrated that the virulence of the MR766 strain in IFNAR1 mice had decreased, showing that the mutation at position 230 in the - M protein is a molecular determinant of plaque morphology, growth property and virulence in mice [48].

The quasispecies distribution of a ZIKV strain (ZIKV-SL1602) isolated from a 29-year-old female traveler was investigated. Data obtained from single molecule real time (SMRT) sequencing were aligned to a consensus sequence and 24,815,877 nucleotide sequences were read. Phylogenetic analysis was then performed and each nucleotide was analyzed to characterize the quasispecies composition of this clinical isolate. For each nucleotide position, the frequency of occurrence of each of the bases was determined and 3375 single-nucleotide variants (SNV) were detected. Interestingly, four variants of the quasispecies population were found to be present at a level of more than 1% of the total population. Mutations in the E protein accounted for 4.1% of the variants and other mutations in the non-structural region—8.2% in the NS2, 1.6% in the NS1 and 1.4% in the NS5 were detected. The phylogenetic data analysis also disclosed that ZIKV-SL1602 clustered within the Asian lineage in close proximity to the WNVs currently circulating in America. Every South American isolate was found to share similar ancestry with the French Polynesian isolates. Hence, it can be inferred that the current circulating South American clade stems from the island of French Polynesia [49].

### 3.3. West Nile Virus (WNV)

West Nile Virus was first characterized in 1937 in the West Nile district of Uganda and was taxonomically placed in the genus *Flavivirus* within the *Flaviviridae* family. The virus later appeared in New York in 1999, where it caused 59 hospitalized infections and 7 deaths before its spread to other parts of the USA between 1999–2001 [50]. WNV survives naturally in a mosquito-bird-mosquito transmission cycle involving the *Culex* sp. mosquitoes [51]. 

A small-plaque (SP) variant was picked from a mutant population of WNV isolated from an American crow in New York in 2000. Characterization of this variant in mammalian, avian and mosquito cell lines led to the discovery that the SP variant contained four nucleotides in its genome that differed from the wild-type genome. Two nucleotide changes led to non-synonymous mutations where there was a P54S change in the prM protein and a V61A change in the NS2A protein. Further analysis of the mutations revealed that deletion at the cleavage site of the prM site did not affect virus replication and its release from mammalian BHK cells. However, the progeny of this virus was no longer able to infect BHK cells. A mutation in the prM region of the TBEV was also reported to cause decreased secretion of virus particles with no effect on protein folding. Lower neurovirulence and neuroinvasiveness were reported when mutation A30P occurred in the NS2A region of the isolate. Further sequencing of the isolate showed that most of the small plaque clones initially isolated reverted back to their wild-type sequence at position 625 in the prM region. Remaining isolates reverted at position 3707 in the NS2A region. These findings suggested that the mutation present in the prM region could be responsible for the phenotype of the small plaque. It is probable that the mutation in the NS2A region was responsible for the determination of the plaque size as the mutation in the prM region was sufficient to revert the isolate to the wild-type phenotype [52].

The genetic diversity of WNV in the avian host was also investigated using next-generation sequencing. The aim was to explore whether the genetically homogeneous cloned virus would go through genetic diversification after passages in young SPF chickens and wild juvenile carrion crows. Data collected revealed that the WNV population showed significant heterogeneity diverging from the quasispecies structure of the initial viral inoculum in both animal models. However, in-depth analysis enacted a comparison between the infection model (SPF chicken and wild juvenile carrion crows) to assess the variations in genetic diversity. It was demonstrated that the WNV genetic diversifications varied significantly from the inoculum in crows with 18 genetic variants but exhibited suboptimal levels of diversifications among the chickens with only 3 single nucleotide variants (SNV) being detected. Hence, natural WNV-susceptible avian hosts could provide a selective setting and contributed to genetic diversifications. NGS technologies have enabled the analysis of WNV quasispecies dynamics, leading to a better understanding of the virus and shed some light on its mechanism of pathogenicity [53].

## 4. Togaviridae

**Figure 3:**
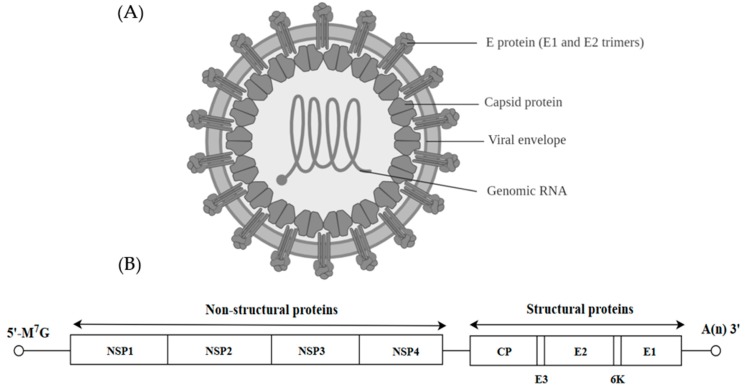
(**A**) The alphavirus structure depicting the position of the E protein (E1 and E2 trimers), capsid protein and the genomic RNA. (**B**) The genome structure of the alphavirus is represented showing the 5′ and 3′ untranslated regions. Open boxes represent the non-structural proteins (NSP1, NSP2, NSP3 and NSP4) and structural proteins (CP, E3, E2, 6K and E1).

Viruses from the *Togaviridae* family can be further classified into the genus *Alphavirus* and *Rubivirus*. Alphaviruses are anthropod-borne viruses [54] and they formed icosahedral particles of about 70 nm with a lipid envelope (Figure 3A) [55]. The spikes of the virion are made up of E1 and E2 glycoproteins organized in a T4 icosahedral lattice of 80 trimers. The alphavirus virion carries a positive single stranded RNA of approximately 11–12 kb as the genetic material [54]. The RNA has a 5′-methylated nucleotide cap and a polyadenylated 3′ end. The viral genome is translated into three structural proteins (CP, E2 and E1) and four non-structural proteins (NSP1, NSP2, NSP3 and NSP4) (Figure 3B).

### Chikungunya Virus (CHIKV)

Chikungunya virus (CHIKV) is an arthropod-borne virus transmitted to humans by mosquitoes and has caused significant human morbidity in many parts of the world [56]. Chikungunya virus causes an acute febrile illness with high fever, severe joint pain, polyarthralgia, myalgia, maculopapular rash and edema. While the fever and rash are self-limiting and are able to resolve within a few days, arthralgia can be prolonged from months to years [57,58]. Some cases of CHIKV disease were associated with neurological complications [59]. The virus has been associated with frequent outbreaks in tropical countries of Africa and Southeast Asia and also in temperate zones around the world. A major outbreak in 2013 affected several countries of the Americas, involving approximately 2 million people [60].

The original geographical distributions of the CHIKV indicated that there are 3 distinct groups and phylogenetic analysis confirmed the West African, the Asian and the East/Central/South African (ECSA) genotypes. The ECSA virus with an A226V substitution in the *E2* envelope gene had caused multiple massive outbreaks in various regions starting in the La Reunion Islands in 2005. The virus then spread to Asia and caused over a million cases in the following years [61,62,63]. The Asian genotype started invading the Americas in 2013, causing massive outbreaks in various countries in Central, South America and the Caribbean. The ECSA virus is now the dominant virus all over Africa and Asia and the Asian genotype is the dominant virus in the Americas [62,64,65,66,67]. Even though a number of CHIKV vaccine candidates are being developed, no effective vaccine is currently available for clinical use [68].

Similar to other RNA viruses with extensive mutation rates, CHIKV produces populations of genetically diverse genomes within a host. Up to date, the role of several of these mutations and the influence of disease severity in vertebrates and transmission by mosquitoes have been studied. Riemersma et al. investigated the intra-host genetic diversity of high and low-fidelity CHIKV variants using murine models. Both the high and low fidelity variants were expected to lower the virulence of CHIKV as compared to the wild-type (CHIKV-WT). However, the high-fidelity variant caused more acute levels of infection such that the onset of the swelling in the footpad exhibited earlier than the CHIKV-WT at 3- and 4-days post-infection (dpi). Moreover, the high-fidelity CHIKV (CHIKV-HiFi) infected mice also displayed higher peaks of disease severity when compared to the CHIKV-WT 7 dpi. This enhanced diversification was subsequently reproduced after serial in vitro passages. In high-fidelity variants, nsp2 G641D and nsp4 C483Y mutations increased CHIKV virulence in the adult mice. The NGS data showed that the CHIKV-HiFi variant produced more genetically diverse populations than the CHIKV-WT in mice. However, the low-fidelity variant gave rise to reduced rates of replication and disease [69].

Plaque size is a common feature of viral characterization. Primary isolates of CHIKV containing variants with different plaque sizes were previously reported [70,71]. Viral variants with different plaque morphology such as small and large plaques had been reported in the 2005 CHIKV outbreak isolates [72]. It is curious how small plaque variants with lower fitness were maintained as a natural viral quasispecies. Plausible explanations indicated that the plaque size might not represent the in vivo growth conditions and that cooperation among variants with different plaque sizes might be required for optimal in vivo replication and transmission fitness. Jaimipak et al. reasoned that if the plaque size did not represent the in vivo growth conditions and the small plaque variants had a similar fitness as the large plaque variants, they would be similarly virulent in a murine model. In order to explore the virulence of the small plaque CHIKV variant in vivo, the pathogenicity of the purified small plaque variant of the CHIKV virus isolated from the sera of the patient in Phang-nga, Thailand in 2009, was tested in neonatal mice [73]. The small plaque variant (CHK-S) showed stable homogenous small plaques after 4 plaque purifications. It also grew slower and produced lower titers when compared with the wild-type virus. After 21 days of infection in the suckling mice with the wild-type and CHK-S variants (injected 103 pfu/mouse), mice which received the CHK-S virus showed 98% survival rate while only 74% of mice survived after infection with the wild-type virus. The small plaque variant of CHIKV obtained by plaque purifications exhibited decreased virulence that makes it appropriate to serve as candidates for live-attenuated vaccine development. The CHIKV variant with the small plaque size formed a major subpopulation in the CHIKV primary isolate during multiple passages in C6/36 cells. This is in line with the reduction of virulence in the suckling mice and indicated that the small plaque variant had reduced in vivo fitness. This suggested that replication cycles in mosquito vectors might play an important role in maintaining the small plaque variant in natural infections. The persistence of the small plaque variant CHK-S clone after multiple passages in C6/36 cells showed that the CHK-S variant might be able to outcompete the large plaque variant when infecting the same cell by an unknown mechanism. Alternatively, small and large plaque variants might cooperate in a way that provided a selective advantage for maintaining the small plaque variant [73].

Comparative sequence alignment of large and small plaque variants of CHIKV primary isolates from the Comoros Island showed two amino acid differences in the nsp2 protease and the nsp3 protein. However, these particular amino acid residues were observed in other CHIKV isolates previously [71] and the significance of these specific sequences for the small plaque phenotype becomes uncertain. Comparison of the entire genome of the CHK-S with other small plaque variants could provide a better understanding of the small plaque variants. In addition, investigation of reverse genetics can provide further insights into the role of specific mutations in the virulence of the CHK-S variant [73].

Abeyratne et al. investigated the role of the capsid in CHIKV virulence by studying the nucleolar localization sequence (NoLS) [74]. NoLS is a region in the N-terminal part of the CHIKV capsid protein, between residues 58 and 110 and is rich in basic amino acids [75,76]. Mutations in the NoLS capsid of the CHIKV led to the development of a unique live-attenuated CHIKV vaccine candidate designated as CHIKV-NoLS. 

The P5 CHIKV-NoLS clone remained genetically stable after five passages in Vero cells or insect cells when compared to the CHIKV-WT. Sequence analysis of the P5 CHIKV-NoLS plaques showed that the two plaque variants had no mutations in the capsid protein. A single non-synonymous change in the nucleotide of the capsid caused an alanine to serine substitution at position 101 in the third plaque variant. However, the substitution did not cause any change in the small plaque phenotype or replication kinetics of the CHIKV-NoLS clone after ten passages in vitro [74]. The in vivo study showed that the CHIKV-NoLS-immunized mice were able to produce long-term immunity against CHIKV infection following immunization with a single dose of the CHIKV-NoLS small plaque variant. Attenuation of CHIKV-NoLS through the NoLS mutation is most likely due to the disruption of the replication of viruses after viral RNA synthesis, however, the precise mechanism of reduced viral titer remained unsolved [76]. The NoLS mutation caused a considerable change in the very basic capsid region involving two nucleotides which could affect the structure of RNA binding, assembly of nucleocapsid and interaction with the envelope proteins [77]. Since the CHIKV-NoLS small plaque variant was attenuated in immunized mice and produced sera which could effectively neutralize CHIKV infection in vitro, it could serve as a promising vaccine candidate needed to control the explosive large-scale outbreaks of CHIKV [76].

## 5. Filoviridae

**Figure 4:**
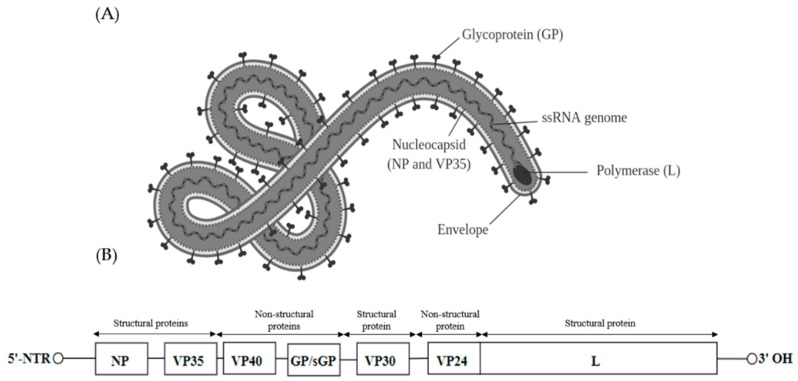
(**A**) Schematic representation of mature Ebola virion consisting of two main components—the nucleocapsid and envelope. The matrix comprising the virion proteins VP24 and VP40 is located between the nucleocapsid and envelope. Glycoprotein (GP) spikes are located on the surface of the envelope (**B**) The genome contains 7 genes which encode the six structural proteins and one non-structural protein [79]. The gene order is 5′-NTR-*NP* (nucleoprotein)-*VP35*-*VP40* (Major matrix protein)-*GP/sGP* (Glycoprotein)-*VP30*-*VP24* (Minor matrix protein)-RNA-dependent RNA polymerase (*l*)-3′-NTR [80].

Viruses found within the *Filoviridae* family can be further classified into five genera—*Marburgvirus*, *Ebolavirus*, *Cuevavirus*, *Striavirus* and *Thamnovirus*. The virions are 80 nm in diameter and appear as branched, circular or filamentous (Figure 4A). Filoviruses contain a linear negative sense single stranded RNA of approximately 19 kb. The genome of the filoviruses encodes for four structural proteins, namely nucleoprotein (NP), RNA-dependent RNA polymerase co-factor (VP35), transcriptional activator (VP30) and a RNA-dependent RNA polymerase (L). There are also three non-structural membrane-associated proteins, namely a spike glycoprotein (GP1,2), a primary matrix protein (VP40) and a secondary matrix protein (VP24) present within the virion membrane [78] (Figure 4B).

### Ebola Virus (EboV)

The *Ebolavirus* genus belongs to the *Filoviridae* family within the order Mononegavirales. Five species have been identified within the genus of *Ebolavirus*—Zaire (EBOV), Bundibugyo (BDBV), Sudan (SUDV), Tai Forest (TAFV) and Reston (RESTV) [81]. Among them, only the Reston virus (RESTV) is assumed to be non-pathogenic for humans. The other four classified as Ebolaviruses are well-known to cause the Ebola virus disease (EVD). The virus causes a severe fever along with systemic inflammation and damage to the endothelial cell barrier, leading to shock and multiple organ failure with high mortality rates in humans and animals [82]. It is transmitted to people from wild animals and spreads in the human population through human-to-human transmission [83]. However, the natural host reservoirs of Ebola viruses are unknown. The average Ebola virus disease (EVD) case fatality rate is around 50%. So far, the largest recorded EVD with 28,652 infections had killed 11,325 people [84]. The Zaire, Bundibugyo and Sudan Ebola viruses are involved in large outbreaks in Africa. 

The glycoprotein (GP) is responsible for cell attachment, fusion and cell entry. The broad cellular tropism of the GP resulted in multisystem involvement that led to high mortality [85]. The Ebola virus has a high frequency of mutation within a host during the spread of infection and in the reservoir in the human population [86]. Alignment of the Glycoprotein (GP) sequences of 66 Ebola virus isolates from the previous outbreaks (old Ebola outbreak of 1976 to 2005) with the new Ebola outbreak isolates (2014) showed some differences in the positions and frequency of the amino acid replacements. Comparative analysis between the isolates from the old epidemic with the new epidemic isolates showed that 19 out of the 22 amino acid mutations were consistently present in the latter [87].

In addition, nucleotide mutations at positions A82V and P382T were present only in the Ebola virus glycoprotein from the new Ebola epidemic 2014 isolates. Mutation at position W291R was also found only in the 2014 isolate but at a very low frequency of occurrence [87]. Having a large number of mutations from previous outbreaks present with more than 90% frequency in the sequence of the new Ebola epidemic isolates as well as the emergence of new mutations (A82V, P382T and W291R) indicated the presence of viral quasispecies in the population. The high mutation rates found in a RNA quasispecies increased the probability of escape mutations and this could explain the escape of the 2014 viral isolates from neutralizing antibodies elicited by the old Ebola epidemic isolates. The structural analysis of the Ebola virus revealed the strong contribution of these residues in the three-dimensional rearrangement of the glycoprotein and they played an important role in the re-emergence of the new epidemic Ebola isolates in 2014. 

Several studies of the Ebola virus glycoprotein showed that the two mutations at positions A82V and T544I might have caused an increase in viral infectivity in humans [88,89,90,91,92,93]. These two mutations reduced the stability of the pre-fusion conformation of the EBOV glycoprotein. Kurosaki et al. investigated the viral pseudotyping of EBOV glycoprotein derivatives in 10 cell lines from nine mammalian species and the infectivity of each pseudotype. The data showed that isoleucine at position 544 mediated membrane fusion and increased the infectivity of the virus in all host species, whereas valine at position 82 modulated viral infectivity but was dependent on the virus and the host. Analysis via structural modeling revealed that the isoleucine 544 changed the viral fusion. However, the valine 82 residue influenced the interaction with the viral entry receptor, Niemann-Pick C1 [94]. The frequency of these two amino acid substitutions (A82V and T544I) varied between different *Ebolavirus* species.

Dietzel et al. studied the functional significance of three non-synonymous mutations in the Ebola virus (EBOV) isolates from the outbreak in West Africa. Among 1000 sequenced Ebola virus genomes, approximately 90% carried the signature three mutations at positions 82, 111 and 759 of the Ebola virus genome. The impact of specific mutations on the role of each viral proteins and on the growth of recombinant EBOVs was analyzed by recently engineered virus-like particles and reverse genetics. A D759G substitution in proximity to a highly conserved region of the GDN motif in the enzymatically active center (amino acid 741 to 743) of the L polymerase was able to increase viral transcription and replication. On the other hand, a R111C substitution in the multifunctional region of the nucleoprotein which is essential for homo-oligomerization and nucleocapsid formation was found to reduce viral transcription and replication. Furthermore, the A82V replacement in the glycoprotein region was able to enhance the efficacy of GP-mediated viral entry into target cells. The combination of the three mutations in the recombinant Ebola virus affected the functional activity of viral proteins and enhanced the growth of the recombinant virus in the cell culture when compared to the prototype isolate [93]. A pilot epidemiological NGS study with a substantial sample size suggested that high mortality in the host was not changed by these three mutations since the rate of mortality in the overall study was not considerably altered throughout the outbreak [95]. 

Furthermore, Fedewa et al. showed that genomic adaptation was not crucial for efficient infection of the Ebola virus. The genomes were characterized after serial-passages of EBOV in Boa constrictor kidney JK cells. Deep sequencing coverage (>×10,000) confirmed the presence of only one single nonsynonymous variant (T544I) of unknown significance within the viral population that demonstrated a shift in frequency of at least 10% over six serial passages. However, passaging the EBOV in other cell lines, such as HeLa and DpHt cheek cells, showed different mutations in the genomes of the viral population [96]. This brings forth the question as to whether the viral strains of the Ebola virus should be directly isolated from patients in order to determine the quasispecies of the Ebola virus.

## 6. Coronaviridae

**Figure 5:**
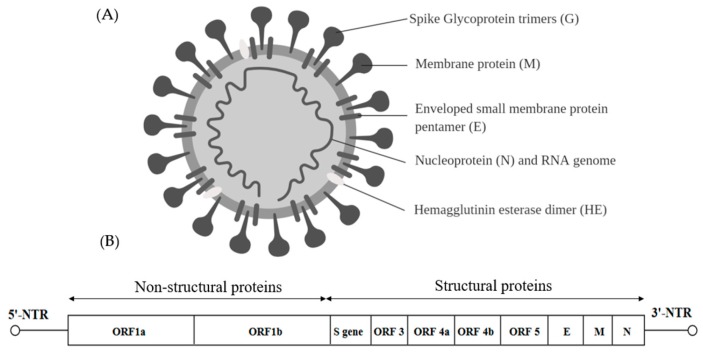
(**A**) Schematic representation of the coronavirus structure and the spike protein features. The virion membrane is enriched with membrane proteins (S, M, E and HE dimer). (**B**) The 31 kb genome and the position of multiple ORFs are illustrated.

Viruses within the *Coronaviridae* family are positive sense, single-stranded RNA viruses capable of infecting three vertebrate classes comprising mammals (*Coronavirus* and *Torovirus*), birds (*Coronavirus*) and fish (*Bafinivirus*). Coronaviruses are the largest RNA viruses identified so far with the enveloped spherical virions of about 120–160 nm and the viral genome is about 31 kb in length (Figure 5A) [97]. The genome consists of many ORFs. Two thirds of the 5′ end is occupied by a replicase gene comprising two overlapping ORFs namely—ORF1a and ORF1b. The four structural proteins are spike glycoprotein (S), small envelope protein (E), membrane glycoprotein (M) and nucleocapsid (N). Accessory regions that are group specific ORFs are designated as ORF3, ORF4a, ORF4b and ORF5 [97] (Figure 5B).

### Middle East Respiratory Syndrome Coronavirus (MERS-CoV)

Middle East respiratory syndrome (MERS) coronavirus is an enveloped, positive-sense, single-stranded RNA virus that was identified for the first time in 2012 in Saudi Arabia. The viral respiratory disease was caused by a novel coronavirus. The causative coronaviruses (CoV) belong to the lineage C of the *Betacoronavirus* within the family *Coronaviridae*. MERS-CoV can infect a broad range of mammals, including humans and is transmitted by the infected dromedary camels [98,99]. Typical MERS symptoms are similar to the common flu but in some patients, pneumonia and gastrointestinal symptoms including diarrhea and organ failure were reported [100]. Since September 2012 to August 2018, 2253 MERS-CoV cases including 840 deaths were reported in 27 countries worldwide [101]. Approximately 35% of patients with MERS-CoV infection have died. 

The nsp1 was reported to suppress protein synthesis by degrading the host mRNA but viral RNA could circumvent the nsp-1 mediated translational shutoff. Terada et al. showed that the double mutations (A9G/R13A) in the non-structural protein 1a (nsp1) affected viral propagation and the plaque morphology. The size of the plaque in the mutated MERS-CoV was smaller and the infectious titers and intracellular viral RNA were decreased in infected Huh7 or Vero cells when compared to the wild-type virus. The formation of the small plaque variant was due to impairment of viral replication via the disruption of the stem-loop (SL) structure of the RNA. In addition, analysis of the biological properties of the nsp1-A9G/R13A mutant showed that the mutant virus possessed low binding activity at the 5′-UTR and promoted translational shutoff against reporter plasmids with or without 5′-UTR [102].

**Figure 6:**
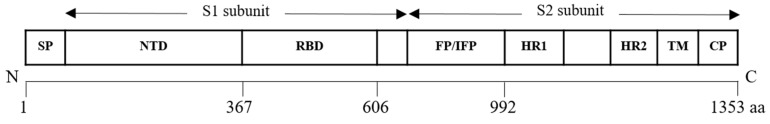
The predicted MERS-CoV spike precursor glycoprotein (encoded by the *S* gene).

Alterations in the coronavirus spike glycoprotein by means of natural and experimentally induced mutations changed cell and organ tropism and virus pathogenicity. The wild-type MERS-CoV spike glycoprotein precursor contains 1353 amino acids arranged into two subunits—an amino-terminal subunit (S1) carrying the receptor binding domain (RBD) and a carboxy-terminal subunit (S2) containing the putative fusion peptide (FP/IFP), two heptad repeat domains (HR1/HR2) and the transmembrane (TM) and intracellular domains (Figure 6).

Lu et al. isolated a diverse population comprising the wild-type and a variant carrying a deletion of 530 nucleotides in the spike glycoprotein gene from the serum of a 75-year-old patient in Taif, Saudi Arabia. The patient subsequently died. Analysis of the MERS-CoV sequence showed an out of frame deletion which led to the loss a large part of the S2 subunit. It contained all the major structures of the membrane fusion in the S2 subunit preceding the early stop codon [103] and this also included the proposed fusion peptide (949–970 aa) [104]. The deletion resulted in the production of a shortened protein bearing only 801 amino acids. In the cell-free serum sample of the patient, mutant genomes with the S530Δ were abundant with an estimated ratio of 4:1 deleted to intact sequence reads. The spike gene deletion would cause the production of a defective virus which was incapable of causing infections or with a lowered rate of infection. Losing the S2 subunit caused a disruption in the membrane holding the spike protein and halted the fusion of the virus to the host. However, in the case of the mutant bearing the S530Δ, the mutation helped to sustain the wild-type MERS-CoV infection by producing a free S1 subunit with a “sticky” hydrophobic tail and the additional disulfide bonds caused the aggregation and mis-folding of proteins. In addition, the mutated S530Δ could form steady trimer complexes that retained biding affinity for the dipeptidyl peptidase 4 (DPP4) and acted as a decoy such that the spike-specific MERS-CoV neutralizing antibodies were blocked.

Park et al. analyzed the non–consensus sequences of MERS-CoV derived from 35 specimens obtained from 24 patients and showed the heterogeneity of MERS-CoV among patients. The maximal level of heterogeneity was recovered from the super-spreader specimens. Moreover, this heterogeneity disseminated in close association with variations in the consensus sequences. It can be inferred that MERS-CoV infections were caused by multiple variants. In-depth analysis of heterogeneity among patients showed a link between D510G and I529T mutations in the receptor-binding domain (RBD) of the viral spike glycoprotein. The two mutations resulted in reduced RBD binding affinity to the human CD26. Moreover, the two mutations were observed to be mutually exclusive, implying that the mutants have the ability to significantly hinder viral fitness. However, variants with D510G and I529T mutations in the S protein demonstrated an increase in resistance against neutralizing monoclonal antibodies and reduced sensitivity to antibody-mediated neutralization [105]. The frequency of each of the single mutant varied greatly but their combined frequency of mutations was elevated in the majority of the samples. Meanwhile, the frequency of the wild type was no more than 10% in the majority of the samples. Therefore, it can be deduced that the selection pressure applied by the host immune response played a crucial part in producing genetic variants and how they interacted with the immune system in humans in MERS-CoV outbreaks [106].

Scobey et al. reported the T1015N mutation in the spike glycoprotein during 9 passages of the virus was able to alter the growth kinetics and plaque morphology in vitro. The mutated MERS-CoV virus (MERS-CoV T1015N) replicated approximately 0.5 log more effectively and formed larger plaques compared to the wild type (MERS-CoV). The data suggested that the mutation T1015N was a tissue culture-adapted mutation that arose during serial in vitro passages [107]. The whole genome sequencing of MERS-CoV revealed the presence of sequence variants within the isolate from dromedary camels (DC) which indicated the existence of quasispecies present within the animal. A single amino acid (A520S) was located in the receptor-binding domain of the MERS-CoV variant. Strikingly, when detailed population analysis was performed on samples recovered from human cases, only clonal genomic sequences were reported. Therefore, the study speculated that a model of interspecies transmission of MERS-CoV whereby specific genotypes were able to overcome the bottleneck selection. While host susceptibility to infection is not taken into account in this setting, the findings provided insights into understanding the unique and rare cases of human of MERS-CoV [108].

## 7. Paramyxoviridae

**Figure 7:**
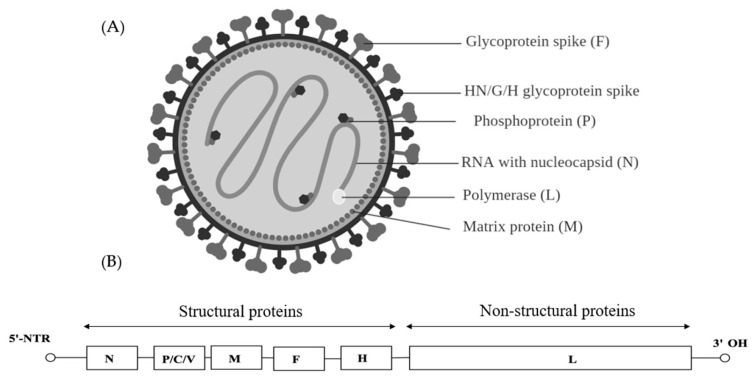
(**A**) A paramyxovirion of 150 nm showing the important viral structural components (F protein, M protein and HN/G/H glycoprotein spikes) (**B**) Paramyxovirus genome containing six genes in the order—*N*, *P*/*C*/*V*, *M*, *F*, *H* and *L*.

*Paramyxoviridae* is a family of viruses in the order Mononegavirales that uses vertebrates as their natural hosts. Currently, 72 species are placed in this family and they are divided amongst 14 genera [109]. Diseases associated with *Paramyxoviridae* included measles (MeV), mumps and Newcastle disease (NDV). *Paramyxoviridae* virions are enveloped and pleomorphic which are presented as spherical or filamentous particles with diameters of around 150 to 350 nm (Figure 7A). The genome is linear, negative-sense single-stranded RNA, about 15–19 kb in length and encode 9–12 proteins through the production of multiple proteins from the *P* gene (Figure 7B) [110]. On the external surface of the virion, glycoproteins possessing hemagglutinin, neuraminidase and cell fusion activities are present. The middle component of the envelope is a lipid bilayer acquired from the host cell as the virus buds off the cytoplasmic membrane. The innermost surface of the envelope is a non-glycosylated membrane protein layer that maintains the outer structure of the virus. The paramyxoviruses can be characterized by the gene order of the viral proteins and by the biochemical characteristics of the proteins associated with viral attachment.

The L protein which is the catalytic subunit of RNA-dependent RNA polymerase (RDRP) is associated with the nucleocapsid protein (N) and phosphoprotein (P) to form part of the RNA polymerase complex. The RNA polymerase complex is covered by the viral envelope consisting of a matrix protein (M) and two glycosylated envelope spike proteins, a fusion protein (F) and cell attachment protein. Cell attachment protein is different based on the genera and it could be hemagglutinin (H in Measles), hemagglutinin-neuraminidase (HN in Mumps and NDV viruses) or glycoprotein G (*Henipavirus*). Some genera within the *Paramyxoviridae* family also contain various conserved proteins including the non-structural proteins (C, NS1, NS2), a cysteine-rich protein (V), a small integral membrane protein (SH) and transcription factors M2–1 and M2–2 [111]. 

Fusion and cell attachment proteins are large glycoprotein spikes that are present on the surface of the virion. Both of these proteins play important roles in the pathogenesis of viruses from *Paramyxoviridae* family and are responsible for attachment to the cellular receptor(s), whereas the F protein mediates cell entry by inducing fusion between the viral envelope and the host cell membrane. The matrix protein organizes and sustains the virion structure. The nucleocapsid associates with genomic RNA and protects the RNA from nucleases. Extracistronic (noncoding) regions include a 3′ leader sequence with 50 nucleotides in length, which works as a transcriptional promoter and a 5′ trailer with 50–161 nucleotides [111].

The genomes of viruses within the family *Paramyxoviridae* are non-segmented and thus cannot undergo genetic reassortment. Like many other RNA viruses, the RNA-dependent RNA polymerase does not have an error proofreading capability and hence many mutations can occur when the RNA is processed. These mutations can build up in the genome and eventually give rise to new variants. Since each protein has an important function, the mutant viruses will exhibit a loss in viral fitness and are eliminated, leaving only those exhibiting good viral fitness [111]. Within the *Paramyxoviridae* family, mutations leading to a spectrum of mutant distributions among Measles virus, Mumps virus and Newcastle disease virus are reviewed.

### 7.1. Measles Virus (MeV)

Measles virus belongs to the genus *Morbillivirus* within the family *Paramyxoviridae* of the order Mononegavirales. Measles is transmitted by air or by direct contact with body fluids. The initial site of viral infection is the respiratory tract, followed by dispersions in the lymphoid tissue, liver, lungs, conjunctiva and skin. The measles virus (MeV) may persist in the brain, causing fatal neurodegenerative diseases. This virus can only infect humans and causes subacute sclerosing panencephalitis and encephalitis [112,113,114]. Measles often lead to fatality in young children (below 5 years) due to complications in respiratory tract infections like pneumonia, brain swelling or encephalitis, dehydration, diarrhea and ear infections [115]. 

The MeV is a negative sense single stranded RNA virus and the genome is composed of six contiguous, non-overlapping transcription units separated by three untranscribed nucleotides. The genes which code for eight viral proteins are in the order of 5′-*N-P/V/C-M-F-H-L*-3′ [116]. The second transcription unit (*P*) codes for two non-structural proteins, C and V, which interfere with the host immune response [117,118,119,120]. 

Early investigations of MeV infections in the HeLa cells with a vaccine-lineage MeV estimated an intra-population diversity of 6–9 positions per genome [121]. This led to the concept that MeV exists as quasispecies in a population. Donohue et al. discovered that MeV was able to adapt and grow in either of the two cellular environments, *viz*. lymphocytic (Granta-519) or epithelial (H358) cells. Passaging the MeV in these two different cell lines resulted in variants exhibiting different replication kinetics. Deep sequencing of the lymphocytic adapted variants demonstrated an increasing number of variants showing mutations within the 11-nucleotide region in the middle of the phosphoprotein (*P*) gene. This sequence mediated the polymerase split and caused an insertion of a pseudo-templated guanosine to the P mRNA, causing a replacement of polymerase cofactor (P) with a type I interferon antagonist (V). The two different variants (lymphocytic and epithelial adapted) had different levels of P and V expressions. It was suggested that the equilibration of the viral quasispecies in the population was based on different V protein expression. Lymphocytic derived MeV variants that exhibited V competent genomes were found at a low frequency for adaptation in epithelial cells. Moreover, a complete wipe out of the V-deficient genomes considerably reduced antiviral innate defense mechanism, suggesting that a good equilibrium of the V and P protein expressions is necessary within the quasispecies population [18].

### 7.2. Mumps

Mumps virus belongs to the genus nus *Rubulavirus* within the *Paramyxoviridae* family of the order Mononegavirales. Mumps is an extremely contagious, acute, self-limited, systemic viral infection that primarily affects swelling of one or more of the salivary glands, typically the parotid glands. The infection could cause pain in the swollen salivary glands on one or both sides of patient face, fever, headache, muscle aches, weakness, fatigue and loss of appetite. Complications of mumps are rare but they can be potentially serious involving inflammation and body swelling in testicles, brain, spinal cord or pancreas. Infections can lead to hearing loss, heart problems and miscarriage. In the United States, mumps was one of common disease prior to vaccination became routine. Then a dramatic decrease was observed in the number of infections. However, mumps outbreaks still occur in the United States and there was an increase in the number of cases recently. Majority of those who are not vaccinated or are in close-contact with the viruses in schools or college campuses are at high risk. There is currently no specific treatment for mumps [122].

The strain Urabe AM9 is one of the mumps virus strains that was widely used in vaccines but this strain was associated with meningitis and was withdrawn from the market. Sauder et al. performed serial passaging of the strain Urabe AM9 in cell cultures and compared the whole nucleotide sequences of the parental (Urabe P-AM9) and passaged viruses (Urabe P6-Vero or Urabe P6-CEF) to investigate the attenuation process and to identify the attenuation markers [123]. Passaging of the Urabe AM9 mumps virus in Vero or chicken embryo fibroblast (CEF) cell lines caused changes in the genetic heterogeneity at particular regions of the genome through either changing of one nucleotide at locations where the starting material showed nucleotide heterogeneity or the presentation of an additional nucleotide to produce a heterogenic site. Virulence of the passaged virus was dramatically decreased in the murine model. Moreover, similar growth kinetics of the virulent Urabe P-AM9 and passaged attenuated variants in the rat brain suggested that the impaired replication ability of the attenuated variants was not the main cause of the neuroattenuation. However, in the rat brain, the peak titer of the neuroattenuated variant was almost one log lower than that of the neurovirulent parental strain. For instance, identical but independent induction of heterogeneity at position 370 of the *F*-gene by substitution of threonine to alanine in passaged virus in Vero and CEF cells suggested a correlation of this mutation to the neuroattenuation phenotype. There was lack of ability to identify heterogeneity for those regions with differences of more than 10% between the detected nucleotides in the consensus sequence. The heterogeneity could be the result of new mutations at these positions or the selection of pre-existing sequences within the minority quasispecies. In addition, passaging of the parental strain in CEF and Vero cells led to the observation of several amino acid alterations in the NP, P, F, HN and L proteins that could affect the virulence of the virus. Thus, the modifications of genetic heterogeneity at particular genome sites could have important consequences on the neurovirulence phenotype. Therefore, extra caution should be exercised in order to evaluate genetic markers of virulence or attenuation of variants based on only a consensus sequence [123].

### 7.3. Newcastle Disease Virus (NDV)

Newcastle disease virus (NDV) belongs to the genus *Avulavirus* in the family *Paramyxoviridae* of the order Mononegavirales. NDV is an avian pathogen that can be transmitted to humans and cause conjunctivitis and an influenza like disease [124]. Clinical diseases affecting the neurological, gastrointestinal, reproductive and respiratory systems are detected in naïve, unvaccinated or poorly vaccinated birds [125]. NDV is a continuous problem for poultry producers since it was identified ninety years ago. It has negatively impacted the economic livelihoods and human welfare through reducing food supplies and many countries were affected since 1926 with NDV outbreaks [126]. 

The NDV genome codes for seven major viral proteins in the order of 5′-*N-P(V)-M-F-HN-L*-3′. In NDV, the hemagglutinin neuraminidase (HN) and fusion (F) glycoproteins are presented on the surface of the virion envelope and contribute to viral infection [127]. The fusion protein is expressed as an inactive precursor (F0) prior to activation by proteolytic cleavage. The cleavage of F0 is crucial for infectivity and works as a key virulence indicator for certain viruses such as virulent strains of avian paramyxovirus 1 (NDV). The F0 cleavage site contains several basic residues which cause the cleavage of the F protein by furin, an endopeptidase present in the trans-Golgi network [110].

NDV strains are categorized based on their pathogenicity in chickens as highly virulent (velogenic), intermediately virulent (mesogenic) or nonvirulent (lentogenic). These levels of pathogenicity can be differentiated by the amino acid sequence of the cleavage site in the fusion protein (F0). Lentogenic NDV strains have dibasic amino acids at the cleavage site whereas the velogenic strains contain polybasic residues. Meng et al. studied the changes in virulence of NDV strains, leading to a switch in lentogenic variant (JS10) to velogenic variant (JS10-A10) through 10 serial passages of the virus in chicken air sacs [128]. However, the lentogenic variants (JS10) remained lentogenic after 20 serial passages in chicken embryos (JS10-E20). The nearly identical genome sequences of JS10, JS10-A10 and JS10-E20 showed that after passaging, both variants were directly generated from the parental strain (JS10). Genome sequence analysis of the F0 cleavage site of the parental strain and the passaged variants revealed that the rise in virulence observed in the parental strain (JS10) stemmed from a build-up of velogenic quasispecies population together with a gradual disappearance of the lentogenic quasispecies. The decline of the lentogenic F0 genotypes of ^112^-E(G)RQE(G)RL-^117^ from 99.30% to 0.28% and the rise of the velogenic F0 genotypes of ^112^-R(K)RQR(K)RF-^117^ from 0.34% to 94.87% after 10 serial passages in air sacs was hypothesized to be due to the emergence of velogenic F0 genotypes. Subsequently, this led to the enhancement of virulence in JS10-A10. The data indicated that lentogenic NDV strains circulating among poultry could lead to evolution of the velogenic NDV strain. This velogenic NDV strain has the potential to cause outbreaks due to the difficulty in preventing contact between natural waterfowl reservoirs and sensitive poultry operations. 

NDV quasispecies comprised lentogenic and velogenic genomes in various proportions. The change in virulence of the quasispecies composition of JS10 and its variants was investigated by analysis of viral population dynamics. The F0 cleavage site was reported to be the main region in which the majority of amino acid changes had occurred and resulting in an accumulation of variants exhibiting velogenic properties due to serial passages. Furthermore, passaging of the virus caused a transition in the degree of virulence of NDV strains from lentogenic to mesogenic and ultimately an increase of the velogenic type. Therefore, NDV pathogenesis could be controlled by the ratio of avirulent to virulent genomes and their interactions within the chicken air sacs and the embryo. The data clearly demonstrated that the status of the quasispecies population is dependent on the pathogenicity of the NDV [128].

Gould et al. reported the presence of the F0 cleavage sequences of ^112^-RRQRRF-^117^ and HN extensions of 45 amino acids in virulent Australian NDV strains [129]. Furthermore, the genome analysis of the avirulent field isolates of NDV puts forth the existence of viruses with virulent F0 sequences without causing obvious clinical signs of the disease [130]. Subsequently, Kattenbelt et al. studied the underlying causes that could affect the balance of virulent (pp-PR32 Vir) and avirulent (pp-PR32 Avir) variants throughout viral infections. The variability of the quasispecies population and the rate of accumulation of mutations in vivo and in vitro were analyzed. The in vivo analysis showed that both virulent and avirulent plaque-purified variants displayed a rise in the variability of quasispecies from 26% and 39%, respectively. The error rate in the viral sequences was observed to increase as well, such that one bird out of three displayed virulent viral characteristics (^112^-RRQRRF-^117^) after passaging of the PR-32 Avir variant. Genome analysis following the in vivo study revealed that a single base mutation occurring in the F0 region led to the switch from RRQGRF to RRQRRF.

On the other hand, in vitro studies showed that the quasispecies distribution of the avirulent isolate harbored 10% of variants bearing the virulent F0 region (RRQRRF). Gene sequence analysis of Australian NDV isolates showed the existence of a novel clade of NDV viruses with the F0 cleavage site sequence of ^112^-RKQGRL-^117^ and the HN region bearing seven additional amino acids. Four field isolates (NG2, NG4, Q2–88 and Q4–88) belonging to the novel clade were propagated for a longer time period in CEF cells prior to sequencing. Analysis revealed the existence of 1–2% of virulent strains with the F0 cleavage site of ^112^-RKGRRF-^117^ in the population [131].

Quasispecies analysis of all the NDV field isolates in this study showed variable ratios (1:4–1:4000) of virulent to avirulent viral F0 sequences. However, these sequences remained constant in the quasispecies population during replication. It was concluded that the virulent strains present in the quasispecies population did not emerge from an avirulent viral population unless the quasispecies population was placed under direct selective pressure, either by previous infection of the host by other avian viruses or by transient immunosuppression [131]. 

## 8. Pneumoviridae

The *Pneumoviridae* family contains large enveloped negative-sense RNA viruses. Previously, this taxon was known as a subfamily of the *Paramyxoviridae* but it was reclassified in 2016 as a family of its own with two genera, *Orthopneumovirus* and *Metapneumovirus*. Some viruses belonging to *Pneumoviridae* family are only pathogenic to humans, such as the human respiratory syncytial virus (HRSV) and human metapneumovirus (HMPV). Human pneumoviruses do not have animal reservoirs and their primary site of infection is the superficial epithelial cells of the respiratory tract. There are no known vectors for pneumoviruses and transmission is thought to be primarily by aerosol droplets [132].

**Figure 8:**
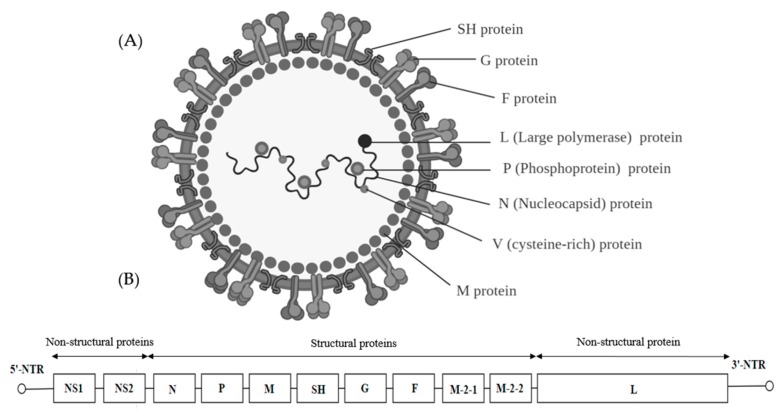
(**A**) Illustration of the pneumovirus structure showing various components of the nucleocapsid and genomic structure (**B**) The negative strand of the unsegmented genome of 13.2–15.3 kb codes for structural proteins such as the nucleocapsid (N) and RNA-dependent RNA polymerase complex (L), phosphoprotein (P), cysteine rich protein (V), the matrix (M) protein, the three surface glycoprotein fusion proteins (F), glycosylated attachment protein (G), short hydrophobic protein (SH) followed by nonstructural proteins such as NS-1 and NS-2 that modulate host responses.

The virions of the pneumoviruses are enveloped with a spherical shape and a diameter of about 150 nm. They have a negative-sense RNA genome of 13 to 15 kb (Figure 8A). The RNA-dependent RNA polymerase (L) binds to the genome at the leader region and sequentially transcribes each gene. The cellular translation machinery translates the capped and poly-adenylated messenger RNA of the virus in the cytoplasm. Members of the genus *Orthopneumovirus* possess 10 genes including NS1 and NS2 which are promoter proximal to the *N* gene. The gene order is *NS1-NS2-N-P-M-SH-G-F-M2-L* (Figure 8B). Alignment of the L proteins showed moderate conservation of the sequences between the human and bovine viruses. Bovine respiratory syncytial virus (BRSV) differs from HRSV in host range and the two viruses bear substantially similar sequences as well as antigenic relatedness [132].

### Respiratory Syncytial Virus (RSV)

Respiratory syncytial virus (RSV) belongs to the genus *Orthopneumovirus* under the family *Pneumoviridae* of the order Mononegavirales. Human respiratory syncytial virus (HRSV) is the primary cause of infection of the upper and lower respiratory tracts with mild, cold-like symptoms in infants and young children. The virus spreads through tiny air droplets. Globally, there are 4–5 million children younger than 4 years with HRSV infections and more than 125,000 are hospitalized every year in the United States. Although the risk of hospital admission is higher in known risk groups such as prematurely born infants. RSV is also responsible for 14,000 deaths in the elderly > 65 years of age annually in the United State [133,134]. On the other hand, bovine respiratory syncytial virus (BRSV) is a common source of pneumonia in calves. Clinical infections stem from yearly outbreaks of the disease during winter and primarily affect calves less than 6 months of age. The target infection site of the viruses are the epithelial layer of the upper and lower respiratory tracts that can damage the bronchioles, leading to severe onset of bronchiolitis in caws [135]. 

Palivizumab (PZ) is the sole humanized monoclonal antibody against an infectious disease that recognizes the fusion protein of respiratory syncytial virus (RSV). Zhao et al. selected a PZ resistant virus by passaging of RSVA2 strain in the presence of PZ in HEp-2 cell culture [136]. Utilization of PZ provided the opportunities to gain new insights into the transmission dynamics and the quasispecies nature of RSV. Protein sequence analysis of a single plaque (MP4) isolated from the fifth passage revealed the substitution of lysine by methionine 272. The mutation caused the cell culture-derived virus to be completely resistant to PZ prophylaxis in cotton rats. Dramatic reduction in replication of the parental strain A2 virus was observed at PZ concentrations ranging from 4 to 40 µg/mL. The replication of the MP4 mutant was not affected by PZ. The growth kinetics of both the parental strain and the variant were almost similar with maximum titers above 10^7^ PFU/mL during the third and fourth day post infection. Hence, it was proposed that the fusion protein supported the entry of the MP4 mutants in HEp-2 cells in an early phase of the replication cycle through a fusion step. The A2 parental strain exhibited limited growth in HEp-2 cells due to its reactivity with PZ. However, the lack of reactivity of the MP4 mutants with PZ suggested that the F1 protein of the MP4 mutant caused a loss of antigenic reactivity with the humanized monoclonal antibody. Preclinical studies in cotton rats predicted the efficacy of PZ in humans. However, the usage of PZ up to 40 µg/mL, especially in immunosuppressed patients, could provide opportunities for the emergence of resistant viruses. Therefore, the PZ resistant viruses in humans could cause the PZ prophylaxis to be ineffective.

Deplanche et al. studied the BRSV and evaluated the genetic stability of BRSV in cell cultures by analyzing the consensus nucleotide sequences of the highly variable glycoprotein G. The BRSV strain W2–00131 was isolated from a calf with respiratory distress syndrome (BAL-T) and was further propagated in bovine turbinate (BT) cells. The genomic region of the BRSV that encodes for the highly variable glycoprotein G showed constant genetic stability for the three variants (3Cp3, 3Cp9 and 3Cp10) after ten continuous passages in BT cells and after in vivo studies [137]. This led to further analysis of the quasispecies population derived from this field isolate. Genomic analysis of more mutants showed that the G-coding region displayed significant variability with mutations ranging from 6.8 × 10^−4^ to 10.1 × 10^−4^ substitutions per nucleotide in vitro and in vivo. 

The majority of the mutations reported previously were present in the W2–00131 RNA populations. A large dominance of non-synonymous over synonymous mutations was observed in all BRSV mutants. The non-synonymous mutations mapped preferentially within the two variable antigenic regions of the ectodomain or close to the highly conserved domain in the G protein [137]. These results suggested that BRSV populations might have evolved as complex and dynamic mutant swarms, despite apparent genetic stability.

Larsen et al. analyzed the nucleotides coding for the extracellular part of the G glycoprotein and the full SH protein of bovine respiratory syncytial virus (BRSV) from several outbreaks from the same herd in different years in Denmark. Identical viruses were isolated within a herd during outbreaks but viruses from recurrent infections were found to vary up to 11% in sequences even in closed herds. It is possible that a quasispecies variant of BRSV persisted in some of the calves in each herd and this persistent variant displayed high viral fitness and became dominant. However, based on the high level of diversity, the most likely explanation is that BRSV was reintroduced into the herd prior to each new outbreak. These findings are highly relevant to understand the transmission patterns of BRSV among calves [138].

## 9. Orthomyxoviridae

**Figure 9:**
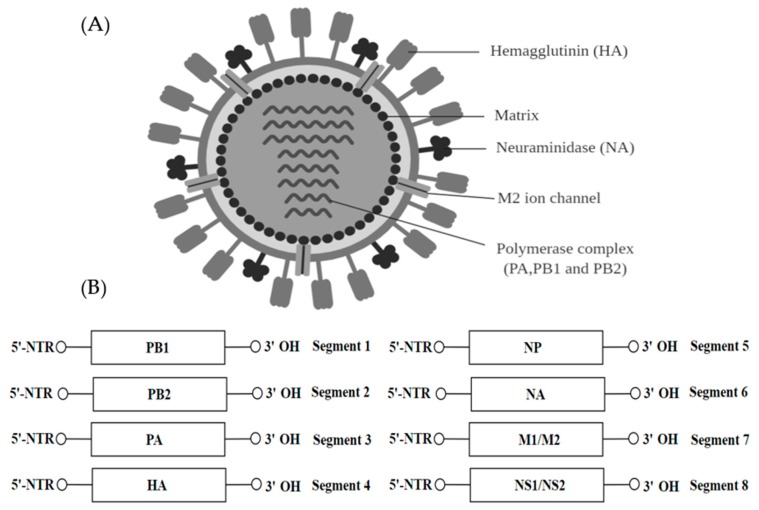
(**A**) The schematic illustration of influenza A virion with the two major glycoproteins displayed on the surface—hemagglutinin (HA), neuraminidase (NA). (**B**) The viral RNA genome of 12–15 kb consists of eight single-stranded RNA segments.

The family *Orthomyxoviridae* belongs to the order of *Articulavirales* and contains seven genera—*Influenza A-D*, *Isavirus*, *Thogotovirus* and *Quaranjovirus*. The virions within the *Orthomyxoviridae* family are usually spherical but can be filamentous, 80–120 nm in diameter (Figure 9A). The influenza virus genome is 12–15 kb and contains 8 segments of negative-sense, single-stranded RNA which encodes for 11 proteins (HA, NA, NP, M1, M2, NS1, NEP, PA, PB1 and PB2) (Figure 9B). Influenza viruses are pathogenic and they can cause influenza in vertebrates, including birds, humans and other mammals [139]. The genome fragments contain both the 5′ and 3′ terminal repeats which are highly conserved throughout all eight fragments.

Orthomyxoviruses employ many different splicing techniques to synthesize their viral proteins while making full use of the coding capacity of the genome. The virion envelope originates from the cell membrane with the addition of one to three virus glycoproteins and one to two non-glycosylated proteins. The viral RNA polymerase (PB1, PB2 and PB3) is involved in the transcription of a single mRNA from every fragment of the genome. The transcription is triggered by cap snatching and the poly(A) tail is added by the viral polymerase stuttering on the poly U sequence. Alternative splicing of the MP and NS mRNA led to the mRNA coding for M2 and NEP proteins. PB1-F2 is translated by leaky scanning from the PB1 mRNA. The structural proteins common to all genera include three polypeptides, the hemagglutinin which is an integral type I membrane glycoprotein involved in virus attachment, the envelope fusion and the non-glycosylated matrix protein (M1 or M) [140].

### Influenza Virus (IV)

The annual influenza epidemics caused about 3 to 5 million cases of severe illness with 290,000 to 680,000 deaths worldwide [141]. Current influenza vaccines have sub-optimal efficacy, as there was a lack of antigenic proximity between the vaccine candidate and the circulating seasonal influenza virus strains. During the 2016–2017 influenza epidemic, the influenza A (H3N2) viruses from the clade 3c.2a were dominant and was associated with severe onset of the disease. The low vaccine efficacy of the 2016–2017 egg-adapted H3N2 (clade 3c.2a) vaccine strain A/Hong Kong/4801/2014 was reported to be due to altered antigenicity [142]. To understand the pathogenesis of A(H3N2) viruses from the 3a.2c clade, it would be of great interest to consider if each infection was being caused by an individual strain or by a swarm of genetically related viruses (quasispecies). This would help to provide an insight into the vaccine coverage and efficacy.

A study investigated the impact of antigenic proximity, genomic substitutions, quasispecies, diversity and reassortment in order to understand the molecular evolution of the influenza A (H3N2) isolated directly from clinical samples. Of the 155/176 whole genomes analyzed, several amino acid substitutions were found to substantially affect the severity of the infection caused by the clade specific viruses. Within the sample, 121 viruses belonged to the genetic clade 3c.2a.1 and eight belonged to 3c.2a2, twenty-four belonged to 3c.2a3, one belonged to 3c.2a4 and one belonged to a different clade 3c.3a. Many distinct substitutions spanning across the whole influenza proteome, HA, NA and non-structural protein 1 were found to be responsible for causing mild and severe disease. Interestingly, two substitutions, V261I and K196E, were found in the NA and the NS1, respectively. These two mutations were found to be particularly significant as they showed the distinction between the strains causing mild and severe infections. Analysis of the clinical isolates showed a difference in a single amino acid residue, 160 K within the HA, whereby 14 cases of glycosylation loss was observed within the quasispecies population linked to severity of infection. Moreover, the degree of diversity within the quasispecies population was reported to be elevated in severe cases when compared to mild ones [143].

The epidemiology and molecular characterization of low and highly pathogenic avian influenza virus strains (LPAIV & HPAIV, respectively) isolated from Germany were investigated. The complete genome analysis of the two strains showed that both LPAIV and HPAIV had high nucleotide similarity with only ten mutations outside the hemagglutinin cleavage site (HACS) which were spread along the six genome segments of the HPAIV. Of the ten mutations, five were previously identified as minor variants in the quasispecies population of the progenitor virus, LPAIV, with 18–42% significant variable frequency [144]. However, studies focusing on the diversity of quasispecies of avian influenza in the human host are few. Watanabe et al. successfully demonstrated that infections caused by a single-virus in vitro produced an evident spectra of mutants in the H5N1 progeny viruses. Analysis of the genetic diversity of the hemagglutinin (HA) revealed that variants with mutated HA had lower thermostability leading to higher binding specificity. Both traits were deemed beneficial for viral infection. On the other hand, other variants with higher thermostability also emerged but were unable to thrive against mutants with lower thermostability [145]. The quasispecies population of influenza A virus was also reported to be in a state of continuous genetic drift in a given subtype population. A viral single nucleotide polymorphism (vSNP) was reported to be important and was shared by more than 15% of the variants within the quasispecies population of the subtype strain in a given season. However, between the season 2010–2011, various vSNPs in the PB2, PA, HA, NP, NA, M and NS segments were shared among variants with more than 58–80% of the sample population and less than 50% of the shared vSNPs were located within the PB1 segment [146].

A study aimed to identify the key mechanisms contributing towards co-pathogenesis of BALB/c mice infected with the A(H1N1) quasispecies. It was revealed that the co-evolution of the quasispecies brought about a complex response due to different expressions of the biphasic gene. A significant upregulation of the Ifng was associated with an increased majority of mutants expressing a differentially expressed gene (DEG) named *HA-G222* gene. This correlated with the increased levels of pro-inflammatory response observed in the lungs of the mice infected with the quasispecies A(H1N1) [147]. Serial passages of the H1N1 virus was also carried out prior to the analysis of its sequential replication, virulence and rate of transmission. Sequence analysis of the quasispecies in the viral population revealed that from the ninth passage onwards, the presence of five amino acid mutations (A469T, 1129T, N329D, N205K and T48N) in the various gene segments (*PB1*, *PA*, *NA*, *NS1* and *NEP*) was detected. Furthermore, mutations located within the HA region indicated that the genetic makeup of the viral quasispecies was distinctly different in the upper and lower respiratory tracts of the infected pigs [148].

## 10. Hepadnaviridae

**Figure 10:**
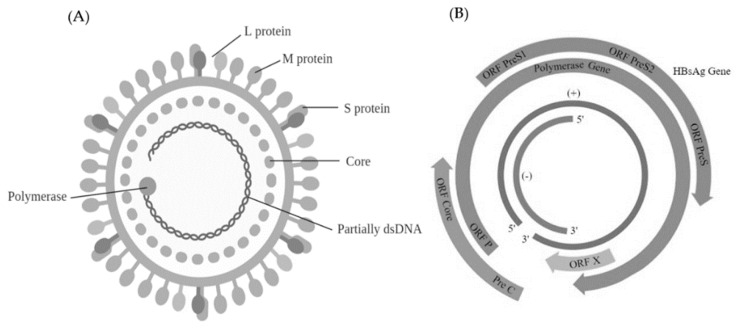
(**A**) Schematic representation of the hepadnavirus structure bearing common structural proteins are portrayed together with the DNA genome (**B**) The overlapping ORFs, negative sense viral RNA and positive RNA strand.

Hepadnaviruses can be found within the family *Hepadnaviridae*. They are further classified into two genera—the mammalian genus *Orthohepadnavirus* and the avian genus *Avihepadnavirus* [149]. These viruses are spherical with 42–50 nm diameter and replicate their genomes with the help of a reverse transcriptase (RT) (Figure 10A). The approximate size of the DNA genome is 3.3 kb with a relaxed circular DNA (rcDNA) supported by base pairing complementary overlaps [150]. The DNA genome is made up of four partly or completely overlapping ORFs that encode for the core protein (Core and preCore), surface antigen protein (PreS1, PreS2 and S), the reverse transcriptase (Pol protein) and the X transcriptional transactivator protein [151] (Figure 10B). Replication occurs by reverse transcription of the progenitor RNA by the RNA polymerase II from the covalently close circular form of the HBV DNA [152].

Complementary to the viral mRNA is a full length negative strand whereas the positive strand is of varied length. The 5′-NTR of the negative strand DNA is covalently attached to the terminal protein (TP) domain of the viral DNA polymerase whereas the 5′-NTR end of the positive sense DNA has a 5′-capped oligonucleotide primer covalently attached. The 3′-NTR of the positive strand ends at a variable position in different molecules and creating a single stranded gap [150].

### Hepatitis B Virus (HBV)

Hepatitis B virus (HBV) is the prototype of hepadnaviruses. It infects humans and can be classified into 8 genotypes. More than one billion people have contracted hepatitis B virus (HBV) and more than 200 million patients are chronically infected with hepatitis B (CHB) [153]. CHB infections result in the development of hepatocellular carcinoma and chronic liver failure [151] and every year CHB causes 880,000 deaths worldwide [153]. Analysis of the immunodominant motifs of the HBV core region from the amino acids 40 to 95 indicated that the positions exhibiting peak rates of variability were found in the main core epitopes, thereby confirming their role in stimulating the immune system. Moreover, the distribution of the variability was observed to occur in a genotype-dependent manner. For instance, HBV isolated from genotype A had higher variability within the core epitope regions but no significant differences in genotype D were observed in the core epitopes and other positions. Further sequential analysis of the samples put forth the dynamic nature of the HBV quasispecies whereby a strong selection for a single baseline variant was linked to a lower variability within the core region pre- and post-treatment. Leucine (L) at position 76 was determined to be the most highly conserved residue and the role of this amino acid was assessed by substitutions of Valine (V) or Proline (P) at position 76. Proline at position 76 was shown to drastically lower the production of Hepatitis B core antigen protein (HBsAg), likely due to the chemical and physical properties of the amino acid. However, substitution with Valine (V) at a similar position brought about a four-fold increase in the Hepatitis B e antigen protein (HBeAg) production when compared to Leucine at position 76. The decrease in the variability observed was associated with a stable quasispecies population after positive selection of the variant exhibiting high fitness level [154].

Although a significant number of RNA viruses demonstrated the existence of quasispecies in their populations due to their low-fidelity polymerases, the phenomenon of quasispecies has been reported to exist in DNA viruses such as Hepatitis B virus (HBV) that replicates via a RNA intermediate. 

In an attempt to elucidate the link between HBV quasispecies and the role of nucleotide analogues present in the quasispecies population, the heterogeneity and distribution of HBV quasispecies using the RT and S regions as a baseline to document the mutation sites were investigated. The quasispecies for the selected regions was analyzed using 608 sequences. In the RT region, no major differences in the composition and diversity of the quasispecies was identified at the nucleotide or amino acid level in patients who responded well to antiviral therapy and those who did not. Similar findings were observed when the S region was examined. However, sequence analysis within RT and S regions showed that the rtM204V/I resistant mutation was observed across the majority of samples prior to the rescue therapy. Interestingly, the frequency of this mutation was noted to drop six months post treatment. Moreover, 3 out of 5 stop codons consistently observed within the RT and S regions were reported to be associated with nucleotide analogue (NA) resistance and affected the HBsAg reading frame. However, the complexity and diversity of the quasispecies of HBV were similar between the CHB patients responsive to the treatment and those who did not. It can be inferred that the characteristics of the quasispecies in CHB patients at the start of the study was not associated to the various viral responses observed in the cohort. Hence, the RT and S regions might not be adequate sites to monitor the response of CHB patients to the rescue treatment [155].

Another challenge to overcome is to accurately determine the origin and spread of a founder population of the virus. Hence, the discrepancies in the evolution of HBV was investigated. Eight related patients with acquired chronic HBV through mother-to-infant transmission were selected and the viral genomes isolated were analyzed. Sequence analysis indicated that the samples originated from a single source of HBV genotype B2 (HBV-B2) which diverged from a tiny common ancestral pool regardless of the route of acquisition. Between individuals, viral strains obtained from a time point showed evidence that they originated from a small pool of the previous time point. This conferred the strain an advantage over other strains with regards to the recovery of the founder state shortly after transmission to the new host and the adaptation to the local environment within the host. Natural selection rather than genetic drift was hypothesized to be the root cause for the evolution of HBV, due to the observed varying patterns of divergence at synonymous and non-synonymous sites. This was in line with the higher rate of substitutions within the host rather than between hosts. Approximately 85/88 amino acid residues changed from common to rare residues. Since these changes were shown not to be a random process, it is concluded that the HBV was able to evolve and change but was limited to a defined range of phenotypes. It can be argued that the mechanism observed thus far suggest that the adaptive mutations accumulated in one individual would not be maintained in another individual and might revert after transmission. Hence, within the host, substitutions were higher than between hosts [156].

Sera collected from two remote rural communities of Nigeria showed that 11% of the population were actively infected with HBV despite limited contacts with other populations. The high prevalence of HBV infection suggested that the transmission of the parental strain introduced was very efficient within the two selected communities. Further analysis showed that the HBV variants belonged to either genotype A or E, with the predominant genotype HBV/E, having a higher prevalence of 96.4%. Subsequent analysis of HBV quasispecies from 24 residents showed that each individual was infected with many different HBV variants from genotypes D and G. This added complexity to the circulating population of HBV in each community. A large network of common sequences was observed among individuals of the community and this is considered as proof of transmission. Furthermore, this pattern of HBV transmission was hypothesized to be linked to recurrent infections with multiple HBV variants or widespread superinfection with varying HBV variants. The close link observed thus far between the HBV/E variants and the recurrent sharing of HBV sequences among individuals made it difficult to clearly distinguish between the HBV/E variants. This suggested that the population of variants could be considered as one swarm of HBV evolving in many different hosts. The coalescent analysis also suggested that the related HBV/E variants in the community originated from one individual HBV variant that was prevalent many years prior to the parental strain being introduced in that community [157].

## 11. Conclusions

RNA viruses are responsible for numerous outbreaks of viral infections with substantial levels of fatality. We discussed how genetic variants carrying spontaneous mutations could give rise to diverse plaque morphologies in different RNA viruses. How the specific mutations could affect viral replications and have an impact on the virulence of the plaque variants are reviewed. The existence of quasispecies in the viral RNA populations is also explored. Many of the RNA viruses displayed different plaque morphologies and these variants could have arisen from a genetically diverse quasispecies population. Such diverse quasispecies in a population could be a key contributing factor to elevated levels of virulence exhibited by the RNA viruses. Through an extensive analysis of different plaque variants and quasispecies within a population, this study could shed more light on the evolutionary pattern and virulence of RNA viruses. More intricate in vitro and in vivo examination of the phenomenon of quasispecies and the relationship between plaque size determinants and virulence should be undertaken to reveal if serious infections are caused by a single strain or through the combined action of diverse quasispecies carrying different mutations. This can be a valuable tool to characterize the mechanisms that led to viral evolution and adaptation in a host. Eventually, discovering an answer to these concerns might ultimately help to design effective vaccines against the ever-evolving RNA viruses.
